# Metabotropic Glutamate Receptors in Glial Cells: A New Potential Target for Neuroprotection?

**DOI:** 10.3389/fnmol.2018.00414

**Published:** 2018-11-13

**Authors:** Simona Federica Spampinato, Agata Copani, Ferdinando Nicoletti, Maria Angela Sortino, Filippo Caraci

**Affiliations:** ^1^Department of Biomedical and Biotechnological Sciences, University of Catania, Catania, Italy; ^2^Department of Drug Sciences, University of Catania, Catania, Italy; ^3^Institute of Biostructure and Bioimaging, National Research Council, Catania, Italy; ^4^Department of Physiology and Pharmacology, Sapienza University of Rome, Rome, Italy; ^5^Neuromed, Istituto di Ricovero e Cura a Carattere Scientifico, Pozzilli, Italy; ^6^Oasi Research Institute, Istituto di Ricovero e Cura a Carattere Scientifico, Troina, Italy

**Keywords:** neurodegeneration, metabotropic glutamate receptor, transforming growth factor-β1, apoptosis, neuroprotection

## Abstract

Neurodegenerative disorders are characterized by excitotoxicity and neuroinflammation that finally lead to slow neuronal degeneration and death. Although neurons are the principal target, glial cells are important players as they contribute by either exacerbating or dampening the events that lead to neuroinflammation and neuronal damage. A dysfunction of the glutamatergic system is a common event in the pathophysiology of these diseases. Metabotropic glutamate (mGlu) receptors belong to a large family of G protein-coupled receptors largely expressed in neurons as well as in glial cells. They often appear overexpressed in areas involved in neurodegeneration, where they can modulate glutamatergic transmission. Of note, mGlu receptor upregulation may involve microglia or, even more frequently, astrocytes, where their activation causes release of factors potentially able to influence neuronal death. The expression of mGlu receptors has been also reported on oligodendrocytes, a glial cell type specifically involved in the development of multiple sclerosis. Here we will provide a general overview on the possible involvement of mGlu receptors expressed on glial cells in the pathogenesis of different neurodegenerative disorders and the potential use of subtype-selective mGlu receptor ligands as candidate drugs for the treatment of neurodegenerative disorders. Negative allosteric modulators (NAM) of mGlu5 receptors might represent a relevant pharmacological tool to develop new neuroprotective strategies in these diseases. Recent evidence suggests that targeting astrocytes and microglia with positive allosteric modulators (PAM) of mGlu3 receptor or oligodendrocytes with mGlu4 PAMS might represent novel pharmacological approaches for the treatment of neurodegenerative disorders.

## Introduction

Neurodegenerative disorders, among the most prevalent, devastating and yet poorly treated illnesses are progressive diseases characterized by slow neuronal death. Dysfunction of glutamatergic transmission plays a central role in the pathogenesis of neurodegenerative diseases (Nguyen et al., 2011). Malfunctioning or aberrant expression of glutamate transporters leads in fact to the accumulation of this neurotransmitter followed by over-activation of ionotropic glutamate receptors, mainly NMDA receptors, a primary event in the pathophysiology of neuronal damage. Activation of NMDA and/or AMPA receptor lacking the GluR2 subunit (Dugan and Choi, 1994; Zipfel et al., 2000), leads to an excessive influx of extracellular Ca^++^ that triggers a cascade of events leading to apoptotic and necrotic death. This occurs both in acute and chronic neurodegenerative conditions such as AD, ischemia, ALS (Doble, 1999; Hardingham and Bading, 2003).

The underlying context is a condition of neuroinflammation, defined as an innate immunological response of the nervous system, involving glial cells, microglia, astrocytes, and cytokines, chemokines, ROS, and other factors they release (Kim and de Vellis, 2005; Block and Hong, 2007; Benatti et al., 2016). Excitotoxicity and neuroinflammation are strictly interconnected since increased extracellular levels of glutamate critically favor activation of glial cells and promotion of neuroinflammatory phenomena in the brain (Olmos and Llado, 2014). In this scenario, glial cells (astrocytes, microglia, and oligodendrocytes) reciprocally interact to contribute to the pathophysiology of neurodegeneration. Under physiological conditions, astrocytes play a key role in the homeostatic control of CNS environment, by removing glutamate from the extracellular space through specific transporters, GLAST and GLT1 (Oliet et al., 2001), as well as by controlling formation (Ullian et al., 2001) and pruning of synapses in response to changes of neuronal activity (Stevens et al., 2007). Dysfunction of astrocytes causes glutamate accumulation with ensuing excitotoxicity (Werner et al., 2001). Reactive astrocytes can further precipitate neuroinflammation (Verite et al., 2018) through the release of pro-inflammatory cytokines and chemokines, including CCL2, which recruits peripheral monocytes into the CNS. Accordingly, apoptotic astrocytes and reactive astrogliosis critically contribute to neurodegenerative processes in different forms of dementia (Heneka et al., 2010) including AD (Kobayashi et al., 2002), vascular (Tomimoto et al., 1997), and frontotemporal dementia (Martin, 2000).

Microglial cells are professional phagocytes (Gomez-Nicola and Perry, 2015) that regulate synapses pruning (Schafer et al., 2012) and phagocytosis of cells undergoing programmed death, both during development and in the mature healthy brain. They also support immune surveillance in the CNS (Zabel and Kirsch, 2013). In response to a prolonged inflammatory stimulus or to the accumulation of misfolded proteins, such as aggregated Aβ, α-synuclein, mutant huntingtin, SOD1, hyperactivated microglia can amplify neurodegeneration, by releasing pro-inflammatory cytokines (Block and Hong, 2007; Mosher and Wyss-Coray, 2014; Streit and Xue, 2014) and ROS (Wilkinson and Landreth, 2006; Dewapriya et al., 2013). Microglia also strongly influence glutamatergic transmission by regulating the expression of glutamate receptors and transporters in neighbor cells (Aronica et al., 2005a; Pickering et al., 2005; Tilleux et al., 2007). Increased extracellular levels of glutamate under pathological conditions, induce microglia chemotaxis to the injury site, through activation of both ionotropic and mGlu receptors expressed in microglia cells (Liu et al., 2009).

In addition to astrocytes and microglia, oligodendrocytes have an essential role in maintaining CNS homeostasis by supporting neuronal myelination and protecting axonal membrane (Rosenbluth, 2009; Bakiri et al., 2011; Harris and Attwell, 2012). Oligodendrocyte dysfunction is mainly involved in the pathogenesis of classical demyelinating diseases (MS and NMO) and leukodystrophies (Fellner and Stefanova, 2013; Ettle et al., 2016). Recent studies suggest that ischemic insults, trauma, and accumulation of abnormal protein aggregates (i.e., α-synuclein, tau, PrP) also cause oligodendrocytes malfunction, leading to myelin disruption and thus neuronal conduction impairment, as reviewed in Ferrer (2018).

## mGlu Receptors in Glial Cells: Distribution and Function

As stated above, glutamate, through the activation of ionotropic receptors, plays a central role in the onset of excitotoxicity. Glutamate activates also a class of G-protein coupled receptors, mGlu receptors, that form a family of eight subtypes (mGlu1 to mGlu8) subdivided into three groups on the basis of their amino acid sequence, G-protein coupling, and pharmacological profile. Group I includes mGlu1 and mGlu5 receptors, which are coupled to G_q_/G_11_ and are functionally linked to polyphosphoinositide hydrolysis and negatively coupled with K^+^ channels (Abdul-Ghani et al., 1996; Nicoletti et al., 2011). Group II (mGlu2, mGlu3) and group III (mGlu4, mGlu6, mGlu7, mGlu8) subtypes are coupled to G_i_/G_o_, negatively regulate adenylate cyclase, but can also activate MAP kinase and PI-3-kinase pathways (Iacovelli et al., 2002; Niswender and Conn, 2010; Nicoletti et al., 2011).

mGlu receptors are widely distributed in the CNS, where they are localized at synaptic and extra synaptic levels in neurons and glia. Group I mGlu receptors are generally localized postsynaptically, surrounding ionotropic receptors, and they modulate depolarization and synaptic excitability. Group II and III are mostly expressed at presynaptic level and control the release of neurotransmitters as reviewed in Niswender and Conn (2010), Ribeiro et al. (2017). mGlu receptor subtypes form homo- and heterodimers (Kammermeier, 2012; Yin et al., 2014; Vafabakhsh et al., 2015). In addition, G_i_-coupled mGlu receptors dimerize with other receptors coupled to G_q_ such as 5-HT_2A_, β_1_-adrenergic, and GABA_B_ receptors (Pin and Bettler, 2016). Evidence of functional interactions between mGlu receptors and estrogen receptors in neurons also exists (Spampinato et al., 2012b).

Intracellular signaling triggered by mGlu receptors has been mainly studied in neuronal cells, whereas less is known in glial cells (Gerber et al., 2007). Group I mGlu receptors activate MAP kinase playing a key role in protein synthesis-dependent neuronal plasticity (Gerber et al., 2007; Hellyer et al., 2017). Translation and transcription factors targeted by MAPK cascades following mGlu receptors activation have been well characterized (Gerber et al., 2007). Group I mGlu receptors dependent phosphorylation of JNKs increases transcription mediated by activator protein-1 (Yang et al., 2006), whereas activation of p38 regulates NF-κB (O’Riordan et al., 2006). More detailed analysis has been carried out in glial cells, and specifically in astrocytes, where stimulation of MAPK and PI3K pathways *via* mGlu3 receptor increases the production of neurotrophic factors (Bruno et al., 1998; Caraci et al., 2011; Durand et al., 2017) promoting neuroprotection against different toxic insults (Ribeiro et al., 2017). When moving to group III mGlu receptors, mGlu4 receptor activation in cultured rat neural stem cells results in inhibition of JNK and p38 mitogen-activated protein kinase, which downregulates the expression of procaspase-8/9/3 and reverses the Bcl-2/Bax balance, finally preventing H_2_O_2_-mediated cell death (Zhang et al., 2015). A protective role for mGlu7 receptor has also been recently found in glial cells and it involves the activation of PI3K/Akt and MAPK/ERK1/2 pathways (Jantas et al., 2018).

According to the principles of “ligand bias” and “functional selectivity,” a G-protein coupled receptor can signal *via* a canonical pathway mediated by the Gα subunit and *via* non-canonical pathways (e.g., MAPK activation) mediated by scaffolding proteins such as β-arrestin (Iacovelli et al., 2014; Hathaway et al., 2015). Recent evidence suggests that mGlu receptors associate with β-arrestin in the initiation of intracellular cascades affecting cellular responses (Hathaway et al., 2015; Hellyer et al., 2017). The recruitment of β-arrestin-dependent signaling pathways occurs in response to G-protein coupled Receptor Kinase (GRK)-dependent phosphorylation and it is strictly ligand-dependent (Hellyer et al., 2017). Future studies are needed in astrocytes and microglial cells to assess whether specific ligands with a functional selectivity can exert different effects on intracellular signaling pathways (e.g., MAPK and PI3K) in neuronal and glial cells.

Of note, the expression of mGlu receptors is developmentally regulated. mRNA levels for mGlu1, mGlu2, and mGlu4 receptors are low at birth and increase during postnatal development (Lujan et al., 2005). In addition, the expression of the shorter mGlu5a receptor isoform is higher in prenatal stages, and mainly detected in cortex, hippocampus and subventricular zone, where it colocalizes with neural progenitors (Boer et al., 2010), astrocytes and microglia. In contrast, in mature brain, mGlu5b receptor is the main isoform expressed (Romano et al., 2002; Lujan et al., 2005).

In glial cells, mGlu1, mGlu3, and mGlu5 receptors are found in astrocytes whereas mGlu2, mGlu3, and mGlu5 receptors are expressed in microglial cells. In oligodendrocytes, mGlu1 and mGlu4 are highly expressed (Ribeiro et al., 2017), whereas mGlu5a and mGlu2/3 receptors are present in early developmental stages and downregulated in mature MBP+ oligodendrocytes (Luyt et al., 2003; Deng et al., 2004; Spampinato et al., 2014).

Glial mGlu receptors regulate glial cell proliferation (Ciccarelli et al., 1997), the release of growth factors, cytokines (Ciccarelli et al., 1999; Aronica et al., 2005b), and neurotransmitters including glutamate, ATP and adenosine, which propagate Ca^++^ signaling between astrocytes and other glial cells (Hamilton et al., 2010). Glial mGlu receptors modulate also the activity and the expression of glutamate transporters, thus participating in the regulation of synaptic function (Aronica et al., 2003b; Vermeiren et al., 2005). Glutamatergic system plays a key role in the pathophysiology of chronic pain and in particular in central sensitization (Guida et al., 2015; Hossain et al., 2017) and neurodegenerative processes leading to cognitive deficits (Giordano et al., 2012). Microglial activation significantly contributes to central sensitization and neurodegeneration promoting the transition from acute to chronic pain (Ji et al., 2014; Hossain et al., 2017). According to this scenario mGlu receptors expressed on glial cells (microglia and astrocytes) might exert a key role in the pathogenesis of chronic pain by modulating both glutamate release and neuroinflammatory phenomena (Chiechio, 2016; Palazzo et al., 2017).

## Group I mGlu Receptors

In physiological conditions, the expression of mGlu1 receptor is very low in astrocytes as well as in cultured cortical astrocytes grown in conventional media. In contrast, the expression is higher in reactive astrocytes of ALS spinal cord (Agrawal et al., 1998; Aronica et al., 2001; Anneser et al., 2004).

Expression of mGlu5 in astrocytes is high prenatally, but decreases after birth (Cai et al., 2000; Yang et al., 2012; Iyer et al., 2014). In physiological conditions, the activity of mGlu5 receptor in cortical astrocytes defines the frequency of Ca^++^ oscillations (Bradley and Challiss, 2011) and the release of gliotransmitters (Agulhon et al., 2008; Fiacco et al., 2009). mGlu5 overexpression has been reported in different neurodegenerative disorders (Ribeiro et al., 2017), in particular in reactive astrocytes surroundings Aβ plaques (Shrivastava et al., 2013), spinal cord lesions (Gwak and Hulsebosch, 2005), MS lesion (Geurts et al., 2003), ALS (Aronica et al., 2001), PD (Tison et al., 2016), and in hippocampal astrocytes from Down syndrome patients (Iyer et al., 2014).

Accordingly, *in vitro*, mGlu5 receptor expression occurs as a reactive response: both mRNA and protein levels are induced in astrocytes grown in media enriched with growth factors (FGF, EGF, TGF-β1) (Miller et al., 1995; Balazs et al., 1997), or exposed to Aβ oligomers (Casley et al., 2009; Lim et al., 2013).

mGlu5 receptor actively regulates glutamate transmission, acting as a sensor of extracellular glutamate concentrations and inducing activation of the glial glutamate transporter GLT-1 (Vermeiren et al., 2005). In contrast, after sustained mGlu5 stimulation, both GLAST and GLT-1 activity are reduced (Aronica et al., 2003a). In astrocytes derived from hSOD1-G93A rats, an established model of ALS, increased expression of mGlu5 receptor mRNA is accompanied by reduced GLT-1 activity and enhanced glutamate-induced excitotoxicity (Vermeiren et al., 2006). Similarly, the accumulation of the glial glutamate and the consequent excitotoxicity described in a mouse model of epilepsy have been related to mGlu5 receptor overexpression in hippocampal astrocytes. Accordingly, the mGlu5 receptor antagonist MPEP, attenuates gliotransmission, preventing neuronal death, with no change of synaptic transmission (Ding et al., 2007).

In the AD APPswe/PS1 transgenic mouse model, high expression of mGlu5 receptor has been described in astrocytes surroundings Aβ plaques, associated to Ca^++^ signaling dysregulation and ATP abnormal release (Shrivastava et al., 2013). As previously described for mGlu1 receptor in neurons exposed to an excitotoxic insult (Spampinato et al., 2012a), astrocytic mGlu5 receptor may activate two opposite pathways: on one side, stimulation of phospholipase C, with ensuing increased intracellular Ca^++^ concentrations, may lead to cell death; on the other hand, however, this effect could be counteracted by alternative activation of the ERK1/2 pathway, through a Homer-dependent mechanism (Paquet et al., 2013). Interestingly, in cultured cortical astrocytes, inflammatory cytokines reduce the expression of mGlu5 receptor (Aronica et al., 2005c; Berger et al., 2012), suggesting a protective adaptation to prevent excitotoxicity (Berger et al., 2012). Furthermore, pharmacological blockade of mGlu5 in astroglial cells prevents motor neurons excitotoxicity (D’Antoni et al., 2011). The inhibition of mGlu5 receptor activity on astrocytes may contribute to the reduction of an inflammatory state in the CNS. Treatment with the mGlu5 receptor antagonist MPEP prevented in fact astrocytic secretion of the inflammatory cytokines IL-6 and IL-8 (Shah et al., 2012).

In cultured microglia, the expression of mGlu1 receptor is barely detectable (Byrnes et al., 2009), but it has been reported *in vivo* in selected microglia cell populations in MS (Klaver et al., 2013). Similarly, the expression of mGlu5 receptor mRNA is low in cultured microglia compared to astrocytes. However, PET imaging studies in animal models exposed to inflammatory stimuli have shown that mGlu5 receptor activation reduced the inflammatory response (Drouin-Ouellet et al., 2011).

*In vitro*, administration of the non-selective group I agonist DHPG, reduced the number of activated microglia (Farso et al., 2009), while the selective mGlu5 receptor agonist CHPG prevented microglial proliferation induced by LPS (Huang et al., 2018), microglial death induced by OGD (Ye et al., 2017), and the expression of several inflammatory cytokines (Byrnes et al., 2009; Loane et al., 2009; Beneventano et al., 2017). The potential of mGlu5 receptor as a new pharmacological target appears also very interesting in traumatic conditions, such as spinal cord lesions or other traumatic events, where reactive microglia, surroundings the area of the lesion, overexpress mGlu5 receptor. In both TBI models and spinal cord lesions, the delayed CHPG administration, also one month after the traumatic event, reduced the number of reactive microglia and the chronic post-injury inflammation (Byrnes et al., 2012; Wang et al., 2013). In TBI and spinal cord lesion, BBB damage may further activate microglia, due to the access in the CNS of blood-borne proteins such as fibrinogen, that induces microglial phagocytic phenotype and the release of inflammatory cytokines, leading to neurotoxicity (Piers et al., 2011). The BBB in normal conditions prevents the access of fibrinogen and other proteins and immune cells that are present in the blood, but its damage is a common event in traumatic injuries (TBI, SCI), ischemic events and neurodegenerative disorders (Zhao et al., 2015), such as AD, where increased barrier permeability is observed (Spampinato et al., 2017). *In vitro*, exposure of microglia to fibrinogen in the presence of the Glu5 receptor PAM (CDPPB) prevented microglia activation and neuronal toxicity (Piers et al., 2011), further underlying the neuroprotective potential of mGlu5 agonists in reducing neuroinflammation.

(RS)-2-chloro-5-hydroxyphenylglycine may prevent micro-glial activation by releasing BDNF and inducing expression of its receptor Trkb, as observed in BV2 microglia cells (Ye et al., 2017). Recently it has been demonstrated that microglia, as many other cell types, communicate with the neighbor cells through shedding of microvesicles that may represent a cargo for neuromodulators, cytokines, and microRNA (Verderio, 2013). In BV2 microglia cells, CHPG induced an increased release of microvesicles carrying the inflammatory miRNA146a (Beneventano et al., 2017), suggesting a pro-inflammatory role of mGlu5 receptor. It has also been suggested that LPS binds directly to mGlu5 receptor inducing Ca^++^ oscillations and NF-κB activity, while attenuating TNFα production (Liu et al., 2014). All these data suggest that microglial mGlu5 receptor exerts an ambivalent role in inflammation.

The neuroprotective potential of mGlu5 receptor agonist CHPG in reducing microglia-induced neuroinflammation may be limited by the fact that the drug has only partial selectivity, poor BBB penetration, and induces a rapid receptor desensitization (Homayoun and Moghaddam, 2010). mGlu5 receptor PAMs have been investigated as potential therapeutic agents in neurological disorders (Xue et al., 2014). *In vitro*, exposure of microglia to mGlu5 receptor PAMs has demonstrated a better control in comparison to CHPG in preventing microglia activation after inflammatory insults (Xue et al., 2014). *In vivo* administration of the mGlu5 receptor PAM, VU0360172, prevented neuronal loss in a TBI model in mice by reducing microglia-induced inflammation (Loane et al., 2014). An open question for future drug discovery processes in neurodegenerative disorders remains how to reconcile the protective effects observed with mGlu5 receptor antagonists on astrocytes, in different experimental models of neurodegeneration, with the anti-inflammatory action of mGlu5 receptor PAMs on microglia, as reported in TBI (Xue et al., 2014). Furthermore we cannot forget that, in neurons, mGlu5 receptors physically interact with NMDA receptors playing a permissive role in mechanisms of excitotoxic neuronal death (Bruno et al., 2017). Accordingly, selective NAMs of mGlu5 receptors are consistently neuroprotective in models of PD and AD (Bruno et al., 2017).

As already stated, the expression of group I mGlu receptors in oligodendrocytes is stage dependent. mGlu1 receptor is expressed in the somas of GalC+ oligodendrocytes in prenatal ages and during the first two postnatal weeks (P3–P14), while later on mGlu1 receptor is localized exclusively at cell processes. mGlu5 receptor shows a similar distribution, although its expression is lower than mGlu1 and it peaks earlier, at P3–P6. A similar pattern is described in human white matter (Jantzie et al., 2010). Both oligodendrocytes and OPC are very sensitive to glutamate mediated toxicity after hypoxia-ischemia (Deng et al., 2003; Fern et al., 2014) and in MS (Macrez et al., 2016). Activation of mGlu1 receptor by DHPG prevented OPC death induced by kainate (Kelland and Toms, 2001; Deng et al., 2004) and non-excitotoxic agents by maintaining the intracellular levels of glutathione and thus reducing oxidative stress (Deng et al., 2004). mGlu5 receptor activation prevented also staurosporine-induced OPC death (Luyt et al., 2006). Starting from this evidence, selective group I mGlu receptor agonists have been studied in periventricular leukomalacia, a condition characterized by OPC damage, that affects the white matter in premature infants after hypoxia-ischemia (Jantzie et al., 2010). Butt et al. (2017) demonstrated that group I receptor agonists can prevent hypoxia-ischemia-induced oligodendrocyte death at all stages of differentiation. Further studies are needed to establish the role of mGlu1 receptor as a new pharmacological target to prevent oligodendrocyte loss in neurodegenerative disorders such as MS, where OPCs are highly vulnerable to excitotoxic damage (Newcombe et al., 2008).

## Group II mGlu Receptors

Group II includes mGlu2 and mGlu3 receptors, which are coupled to G_i_/G_o_ proteins and have been recently studied as a relevant pharmacological target in neurodegenerative disorders (Bruno et al., 2017). Both mGlu2 and mGlu3 receptors are preferentially localized in the pre-terminal region of axon terminals, where they negatively regulate neurotransmitter release. Only mGlu3 receptor is expressed in astrocytes and is present at all developmental stages (Sun et al., 2013), whereas microglial cells express both mGlu2 and mGlu3 receptors (Geurts et al., 2003). mGlu2/3 receptors levels increase in astrocytes in response to FGF and EGF (Aronica et al., 2003a) and after exposure to pro-inflammatory cytokines (TNFα and IL-1β) (Berger et al., 2012). mGlu3 receptor actively participates in the control of extracellular glutamate by increasing the expression of GLAST and GLT-1 (Gegelashvili et al., 2000; Aronica et al., 2003a; Yao et al., 2005; Zhou et al., 2006). Hence, the use of mGlu3 receptor agonists and/or PAMs has been proposed in the treatment of ALS in which a defect of GLT-1 has been well described (Rothstein et al., 1995; Battaglia et al., 2015). In addition, astrocytic mGlu3 receptors, through activation of MAPK and PI3K pathways, lead to neuroprotection by increasing synthesis and secretion of neurotrophic factors (Bruno et al., 2017), among others, TGF-β1, that prevents both NMDA- and Aβ-induced toxicity on neurons (Bruno et al., 1998; Corti et al., 2007; Caraci et al., 2011) and GDNF. The latter is an established neurotrophic agent for nigral dopaminergic neurons, and has shown neuroprotective and restorative activity in a variety of preclinical models of parkinsonism (Ibanez and Andressoo, 2017). It also protects cultured spinal motor neurons from excitotoxicity (Battaglia et al., 2015). Pharmacological activation of mGlu3 receptor in mice increases GDNF mRNA and protein levels in striatal neurons (Battaglia et al., 2009). Hence, selective mGlu3 receptor enhancers may be effective in slowing neuronal degeneration in different conditions such as ALS (Battaglia et al., 2015) and PD (Bruno et al., 2017).

In this regard, a glial-neuronal interaction mediated by astrocytic mGlu3 receptors seems to play a critical role. Early studies have shown that mGlu2/3 receptors agonists protect cortical neurons against excitotoxic death only in the presence of astrocytes (Caraci et al., 2012; Bruno et al., 2017). Studies carried out in cultured astrocytes from mGlu3(-/-) mice (Corti et al., 2007; Caraci et al., 2011; Battaglia et al., 2015) have clearly demonstrated the key role of astrocytic mGlu3 receptor in mediating the neuroprotective effects of mGlu2/3 receptor agonists. Activation of mGlu3 receptor activity also protects astrocytes from OGD (Ciccarelli et al., 2007) and nitric oxide damage, due to the reduction of cAMP content and consequent activation of PI3K/Akt pathway (Durand et al., 2010, 2013).

mGlu3 receptor might represent a relevant pharmacological target to develop disease-modifying drugs in AD (Caraci et al., 2018a). Although no clear data are available in human AD brains, mGlu3 receptor expression appears reduced in several animal models of AD (Dewar et al., 1991; Cha et al., 2001; Durand et al., 2014; Knezevic and Mizrahi, 2018). When treated with the mGlu2/3 receptor agonist LY379268, astrocytes *in vitro* reduced neuronal Aβ toxicity through the release of neuroprotective factors such as TGF-β1 (Caraci et al., 2011) and BDNF (Durand et al., 2017). TGF-β1 is known to exert anti-inflammatory and neuroprotective effects in experimental models of AD (Chen et al., 2015), and stimulates Aβ clearance by microglia (Tichauer and von Bernhardi, 2012). It also exerts a key role in synaptic plasticity and memory formation promoting the transition from early to late LTP (Caraci et al., 2015). A selective deficit of TGF-β1 signaling has been found in an early phase of AD and appears to critically contribute to neuroinflammation and cognitive decline in AD (Caraci et al., 2018b). Rescue of TGF-β1 signaling represents therefore a new pharmacological strategy to yield neuroprotection in AD. Activation of mGlu3 receptor can positively interfere also with other relevant steps of AD pathogenesis by reducing Aβ production (Durand et al., 2014) or increasing Aβ clearance (Durand et al., 2017). Astroglial mGlu3 receptors stimulate the activity of α-secretase, the enzyme that cleaves APP downstream of the N-terminus domain of Aβ_(1-42)_ (Durand et al., 2014). When exposed to LY379268, astrocytes reduce the levels of β-secretase, while increasing the expression of sAPPα, thereby reducing neurotoxic Aβ. Recently, it has been demonstrated that LY379268 can increase Aβ uptake in astrocytes and microglia, finally promoting Aβ removal from the extracellular space (Durand et al., 2017). The contribution of mGlu3 receptor seems equivocal because Aβ phagocytosis was not prevented by LY2389575, a selective mGlu3 receptor NAM, suggesting that the effects observed after LY379268 stimulation can also involve mGlu2 receptor activation (Durand et al., 2017).

Microglia respond to Aβ with increased glutamate release (Barger and Basile, 2001). Exposure of microglial cells to the active fragment Aβ_(25-35)_ induces also mGlu2 receptor activation, that can lead to increased neurotoxicity (Taylor et al., 2002, 2005). Activation of mGlu2, but not mGlu3 receptors, promotes in fact a pro-inflammatory and neurotoxic phenotype that releases TNF-α and FAS-L, and enhanced microglial reactivity in response to chromogranin-A, up-regulated in AD (Taylor et al., 2002, 2005).

An open question remains whether activation of microglial mGlu3 receptor can promote the release of TGF-β1, then contributing to the overall neuroprotective activity of LY379268 observed in Aβ-treated mixed neuronal cultures (Caraci et al., 2011).

It is well known that microglial activation plays a central role in the pathogenesis of MS (Strachan-Whaley et al., 2014). Exposure to myelin fragments induces microglia activation *in vitro*, promoting the release of glutamate and TNF-α, followed by neuronal death. Interestingly, activation of microglial mGlu2 receptor exacerbates myelin-evoked neurotoxicity, whilst activation of mGlu3 receptor is protective (Pinteaux-Jones et al., 2008).

Suboptimal neuroprotective effects of orthosteric mGlu2/3 receptor agonists have been observed in animal models of global and focal brain ischemia (Bond et al., 1998; Bond et al., 2000), probably due to the involvement of mGlu2 receptors expressed in neurons (Corti et al., 2007; Motolese et al., 2015; Mastroiacovo et al., 2017). However, the role of microglial mGlu2 receptor in stroke ischemia has not been fully elucidated. mGlu2 and mGlu3 receptors are expressed by microglia in the ischemic penumbra, where apoptotic neuronal death develops slowly, making this area more amenable to therapeutic intervention. Microglial cells mediate neurotoxicity in the stroke penumbra (Kaushal and Schlichter, 2008) and in experimental models of ischemia, it has been demonstrated that glutamate, released by “ischemic” neurons, activates microglia through group II mGlu receptors with the following activation of NF-κB, induction of TNF-α, and subsequent neuronal death (Kaushal and Schlichter, 2008). New studies should be conducted in cultured microglia from mGlu2(-/-) mice to better understand the role of microglial mGlu2 receptor in the pathophysiology of stroke ischemia.

## Group III mGlu Receptors

The function of group III mGlu receptors in astrocytes has not been fully explored. They are almost undetectable in gray matter of normal human brains (Blumcke et al., 1996; Tang and Lee, 2001), although the expression of mGlu4 receptor, and occasionally of mGlu8 receptor, was described in reactive astrocytes surrounding MS lesions (Geurts et al., 2005) as well as in other pathological conditions (Tang and Lee, 2001; Aronica et al., 2003b). The expression of mGlu4 receptor in astrocytes cultured *in vitro* is still debated. Some studies, but not others (Ciccarelli et al., 1997), reported the expression in primary cortical cultures (?), and induction after exposure to LPS (Spampinato et al., 2014). In contrast, mGlu7 receptor subtype is not expressed in glial cells (Ciccarelli et al., 1997; Aronica et al., 2001; Taylor et al., 2003). Of note, stimulation of mGlu7 and mGlu8 receptors may have a role in the differentiation of progenitor cells in the ventral midbrain (Vernon et al., 2011). Stimulation with the group III mGlu receptor agonist L-AP4 reduces in fact the proliferation of fetal mouse neocortical progenitor, and promotes their differentiation toward an oligodendrocytic and astrocytic phenotype (Nakamichi et al., 2008).

One of the principal effects exerted by mGlu4 receptor agonists is the reduction of the inflammatory response. The expression of the chemoattractant chemokine Rantes (CCL5), whose role in neuroinflammation has been well documented (Sorensen et al., 1999), was significantly downregulated when astrocytes were exposed to inflammatory cytokines in the presence of L-AP4 (?). This *in vitro* evidence was supported by reduction of the disability score in mice with experimental autoimmune encephalomyelitis treated with l-AP4 (Besong et al., 2002). In astrocyte and oligodendrocyte co-cultures, L-AP4 prompted astrocytic release of TGF-β1, preventing kainate-induced cell death in oligodendrocytes (Spampinato et al., 2014). In contrast, L-AP4 direct treatment on oligodendrocytes was not able to prevent kainate-induced toxicity, but accelerated the differentiation of OPC into mature MBP+ and fully branched oligodendrocytes (Spampinato et al., 2014).

Acting on astrocytes, group III mGlu receptors may also improve glutamate uptake, modulating the expression of both GLT-1 and GLAST. Zhou et al. (2006) reported that L-AP4 prevented neurotoxicity of LPS-treated astrocytes, an effect likely mediated by the increased expression of glutamate transporters. Similar effects were reported in astrocytes exposed to MPTP in the presence of mGlu4 receptor agonists (Yao et al., 2005). In conditions of energy failure, e.g., ischemia, GLT-1 may act paradoxically, running in a reverse mode and thus aggravating the load of glutamate (Rossi et al., 2000; Bonde et al., 2003). Under these conditions, stimulation of mGlu4 receptor may prevent GLT-1 upregulation in reactive astrocytes, thus reducing the aberrant glutamate transport and contributing to neuroprotection (Rodriguez-Kern et al., 2003).

In cultured microglia, the expression of mGlu4, 6 and 8 receptors has been clearly reported (Taylor et al., 2003). In MS patients, mGlu8 receptor was described in the microglial/macrophage line, in particular in the parenchyma and perivascular cuff (Geurts et al., 2003). The overexpression of the receptor in these areas may be induced by the presence of specific cytokines and growth factors released by the environment surrounding the lesions.

As reported (Taylor et al., 2003), agonists acting on group III mGlu receptors prevent microglia activation *in vitro*. The mechanisms involved in these processes were not clarified, but the release of trophic factors from microglia (Conn and Pin, 1997), or reduced glutamate discharge (Taylor et al., 2003) could be claimed. Glutamate may in fact act in a negative feedback loop reducing its own release in inflammatory states (McMullan et al., 2012). Further, *in vitro* exposure of microglia to the mGlu4 receptor PAM, ADX88178, reduced the LPS-induced expression of MHCII and iNOS, while reducing the release of TNFα (Ponnazhagan et al., 2016).

mGluR4, for its anatomical distribution and function, seems to be an interesting pharmacological target for the treatment of PD. mGluR4 orthosteric agonists have been tested in neurotoxin-based rat models of PD, where they reduced signs of inflammation and the consequent dopamine neuronal loss (Battaglia et al., 2006; Zhou et al., 2006; Betts et al., 2012). These effects were also observed using more potent, selective and orally bioavailable mGlu4 receptor PAMs, such as ADX71743 (Le Poul et al., 2012). The increasing importance of the potential use of mGlu4 receptor agonists in PD relies in their capability to modulate directly neuronal circuits, and as additive effects, to attenuate pro-inflammatory immune mechanisms associated with PD. Accordingly, VU0155041, a mGlu4 receptor PAM, reduces microglia activation in the substantia nigra pars compacta of 6-OHDA-treated rats (Betts et al., 2012).

## Conclusion

**FIGURE 1:**
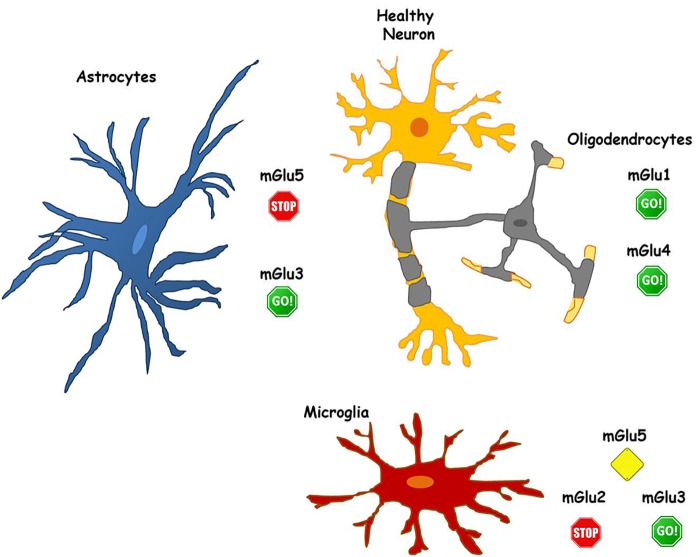
The role of mGlu receptors in different glial cell types. Astrocytes express both mGlu3 and mGlu5 receptors. mGlu3 receptor stimulation initiates mechanisms that induce neuroprotection, while mGlu5 receptor activity promotes neuronal damage. Hence, allowing pharmacological activation of mGlu 3 receptor (GO!) and blocking mGlu5 receptor activity (STOP) in astrocytes could be valuable for the maintenance of neuronal health. Similarly, in microglia, mGlu3 receptor stimulation plays beneficial effects on neurons (GO!), while blockade of mGlu2 (STOP) appears necessary to prevent neurotoxicity. Less defined is the function of glial mGlu5 receptor that, playing a dual role, may be a more complex target for pharmacological intervention (Alert yellow sign). Pharmacological activation of both mGlu1 and mGlu4 receptors, expressed in oligodendrocytes, appear to be neuroprotective (GO! sign).

Metabotropic glutamate receptors are highly and diffusely expressed in glial cells. This, on one side, increases the options for therapeutic interventions, but, on the other side, makes even more difficult the possibility to target selectively single receptors to yield neuroprotection. As mentioned above, different mGlu receptors may give rise to contrasting outcomes when activated in neurons or in glial cells or even in different types of glial cells (see Figure 1).

mGlu5 receptor agonists for instance, might be detrimental for neuroprotection. On neurons, mGlu5 receptor stimulation has been linked to increased synaptotoxicity in AD and PD models (Bruno et al., 2017). A similar potentiation of neurotoxicity is also observed following activation of mGlu5 receptor in astrocytes. Therefore, the anti-inflammatory effects mediated by the activation of mGlu5 receptor on microglia may be vanished by the effects that mGlu5 receptor agonists could exert acting directly on neurons and/or on astrocytes. However, when considering as a whole the different role of mGlu5 receptor in astrocytes and microglia in neurodegenerative disorders, NAMs of mGlu5 receptor should continue to represent a relevant pharmacological tool to develop new neuroprotective strategies in these diseases, with astrocytes as the main target (see Figure 1).

mGlu3 receptor represents a validated pharmacological target to develop disease-modifying drugs in neurodegenerative disorders such as AD, where the development of mGlu3 receptor PAMs might be successful (Figure 1). These drugs acting on receptors expressed in glial cells exert a relevant neuroprotective activity in AD models through multiple mechanisms such as the release of neurotrophic factors (TGF-β1, BDNF) and the reduction of Aβ production (Bruno et al., 2017). More specifically, drugs with mGlu2 NAM/mGlu3 PAM activities might be considered excellent candidates for the treatment of AD. The potential disease-modifying activity of pure mGlu2/3 receptors agonists may be vanished by the detrimental effects of mGlu2 receptor in neurons. Drugs endowed with mGlu2 NAM activity may limit this effect and also cater the potential to restrain microglia-induced neuroinflammation that is consistently found in different neurodegenerative disorders such as AD and PD.

Finally, the effects mediated by mGlu4 receptor expressed either in astrocytes, microglia and oligodendrocytes appear promising for the development of mGlu4 receptor modulators in the treatment of neurodegenerative disorders (Figure 1). In this regard, the possibility to prevent neuroinflammatory phenomena with mGlu4 PAMs seems particularly intriguing since the effect exerted on glial cells may be synergized by the modulatory activity shown by mGlu4 receptor agonists on the peripheral immune system (Fallarino et al., 2010; Fazio et al., 2014, 2018).

Moving from the evidence discussed in the present review, we believe that targeting astrocytes and microglia with mGlu3 PAM or oligodendrocytes with mGlu4 PAMs might actually represent a novel pharmacological approach for the treatment of neurodegenerative disorders.

## Author Contributions

SFS, MAS, and FC wrote the paper. AC and FN contributed to write the paper and revised it critically for important intellectual content.

## Conflict of Interest Statement

The authors declare that the research was conducted in the absence of any commercial or financial relationships that could be construed as a potential conflict of interest.

## Abbreviations

6-OHDA: 6-hydroxydopamine
AD: Alzheimer disease
AMPA: α-amino-3-hydroxy-5-methyl-4-isoxazolepropionic receptor
ASL: amyotrophic lateral sclerosis
Aβ: beta amyloid
BBB: blood brain barrier
BDNF: brain derived neurotrophic factor
CHPG: (RS)-2-chloro-5-hydroxyphenylglycine
CCL2: monocyte chemoattractant protein-1
CNS: central nervous system
DHPG: (S)-3,5-dihydroxyphenylglycine
EGF: epidermal growth factor
FGF: fibroblast growth factor
GalC+: galactocerebroside
GLAST: L-glutamate/L-aspartate transporter
GLT-1: glutamate transporter-1
IL-6: interleukin 6
JNK: c-Jun N-terminal kinase
L-AP4: L-(+)-2-amino-4-phosphonobutyric acid
LPS: lipopolysaccharide
LTP: long term potentiation
MBP: myelin basic protein
mGlu: metabotropic glutamate receptor
MPEP: 2-methyl-6-(phenylethynyl)pyridine
MS: Multiple sclerosis
NAM: negative allosteric modulator
NMDA: *N*-methyl-D-aspartate receptor
NMO: neuromyelitis optica
OGD: oxygen glucose deprivation
OPC: oligodendrocytes progenitor cell
PAM: positive allosteric modulator
PD: Parkinson disease
PI3K: phosphatidylinositol-3-kinase
ROS: reactive oxygen species
sAPPα: soluble amyloid precursor protein
SCI: spinal cord injury
SOD-1: superoxide dismutase
TBI: traumatic brain injury
TGF-β1: transforming growth factor β1
TNFα: tumor necrosis factor α
TrkB: tyrosin receptor kinase B
